# A Patient with Milky Urine: Nonparasitic Chyluria and Silver Nitrate Sclerotherapy

**DOI:** 10.1155/2020/8853473

**Published:** 2020-07-22

**Authors:** M. Sivashankar, A. C. N. Nandasena

**Affiliations:** Department of Urology, Colombo North Teaching Hospital, Ragama, Sri Lanka

## Abstract

Chyluria has become a rare clinical presentation in Sri Lanka, which may have a direct correlation with the low prevalence of lymphatic filariasis following the use of diethylcarbamazine and albendazole mass drug administration (MDA) for five rounds between 2002 and 2006. Here we report a 50-year-old male who presented with milky urine and progressive weight loss, diagnosed as having nonparasitic chyluria. The patient was initially managed with a trail of diethylcarbamazine (DEC) 6 mg/kg/day for 21 days and a low-fat diet with an unsatisfactory response. Subsequent management with endoscopic instillation of 0.5% silver nitrate brought in him a quick response, which was maintained for a year. Endoscopic sclerotherapy is considered a safer, effective and a minimally invasive treatment option for symptomatic patients.

## 1. Introduction

The presence of intestinal lymph (chyle) in the urine is known as chyluria. From the etiological perspective, chyluria is classified into two types: parasitic and nonparasitic (1). The more common parasitic type is caused by Wucheraria Bancrofti and is found principally in filarial endemic areas, while the nonparasitic type has a variety of etiological factors falling under a broad category (2). Chyluria is still a symptom that urologists encounter, even though Sri Lanka has achieved filariasis eradication status according to WHO criteria (7). This clinical scenario aims to share our experience of a patient with nonparasitic chyluria following a lympho-urinary fistula of the left kidney, who has been treated successfully with endoscopic silver nitrate sclerotherapy.

## 2. Case Presentation

A 51-year-old male was admitted with a complaint of milky urine and significant weight loss (10 kg) for over four months. On further assessment, neither lower urinary tract symptoms nor a contact history of tuberculosis was observed. The patient was cachectic with a body mass index of 20.2 kg/m^2^.

**Figure 1:**
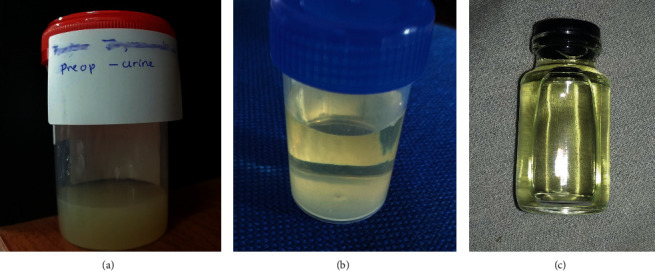
The color of urine samples prior and after the endoscopic sclerotheraphy, (a) prior to sclerotheraphy, (b) post surgery day 5, (c) one month after surgery.

**Figure 2:**
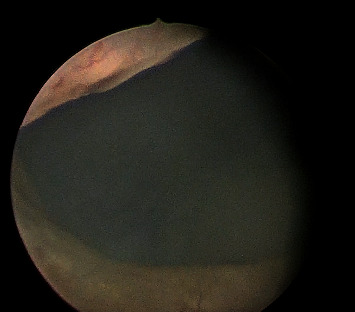
Cystoscopy view showing the chyluria.

**Figure 3:**
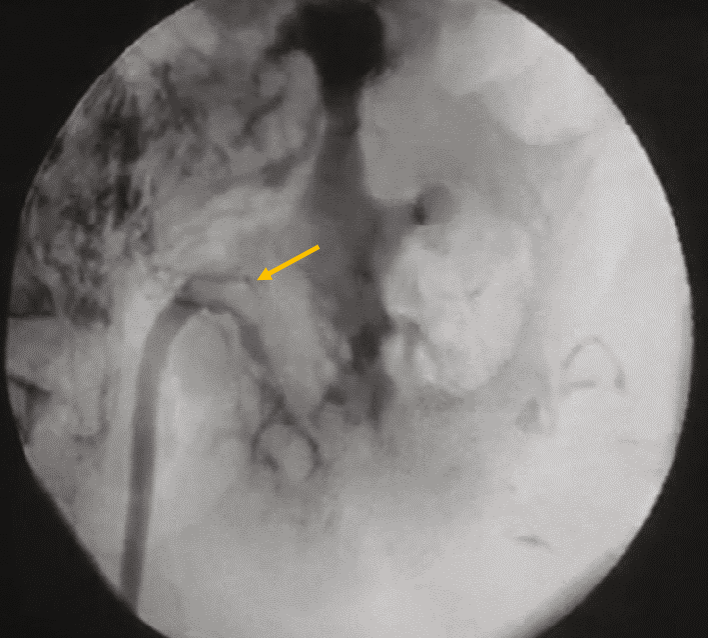
Left side retrograde pyelogram is showing the main lympho-urinary fistula at renal pelvis close to pelvic ureteric junction (marked by arrow).

Macroscopically, urine appeared milky in colours (Figure 1(a)), and the analysis demonstrated severe proteinuria and a high triacylglycerides level of 370 mg/dl (standard 1-10 mg/dl). Chloroform test for chyluria was positive, whereas filarial IgM and IgG antibodies were negative. Imaging studies of the urinary tract, which included an ultrasound scan (KUB) and CT-urography, revealed no abnormality. During diagnostic rigid cystoscopy and ureterorenoscopy, egress of milky urine from the left ureter was observed (Figure 2). The retrograde pyelogram, performed subsequently, demonstrated a left-sided lympho-urinary fistula at the level of the renal pelvis (Figure 3).

Initially, he was managed with a low-fat diet and a trial of diethylcarbamazine 6 mg/kg/day for 21 days with an inadequate response. Persistent symptoms, despite initial management, encouraged us to change over to instillation therapy. 10 ml of 0.5% silver nitrate was instilled endoscopically to the left ureter via a 6 Fr ureteric catheter, and the procedure was repeated for another three cycles at 30 min intervals. Subsequently, bladder irrigation was continued for 12 hours, and cefuroxime was administered intravenously for three days. He had an uneventful recovery with complete clearance of urine over five days (Figure 1(b)) and was discharged later on a low-fat diet. On routine follow up at one month, his urine was completely clear (Figure 1(c)), and a 3 kg increase in body weight was also observed. Follow up was carried out for twenty-two months with no recurrence of symptoms.

## 3. Discussion

Chyle is a milky fluid, rich in lymph and chylomicrons. Normally lymph flows through the intestinal lacteals to the cisterna chyle and the thoracic duct, which drain into the left subclavian vein. Although the pathogenesis of chyluria is not well described, two theories have been postulated: obstructive and regurgitative mechanisms for leakage of lymph into the urine (2). Commonly, leakage resulting from lympho-urinary fistula develops at the level of the renal pelvicalyceal system, but it can even occur at the level of the ureter and bladder (1).

Chyluria is confirmed by simple bedside chloroform or ether test, which extracts fat globules and forms an organic layer leaving the remaining urine clear. Sudan III test is also can be used to confirm fat globules in the urine. However, assessment of triglyceride levels in urine is by far the most accurate test to confirm chyluria when it is more than 15 mg/dl (1, 3).

Ascertaining the etiology, side, and the site of chyluria is an important part of the management. Usually, ultrasound scan (KUB) and computed tomogram imaging of upper tracts would fail to provide the necessary information decisive to the management of chyluria. However, it may reveal an uncommon, incidental primary cause helpful in the management of nonparasitic chyluria. Magnetic resonance imaging might show clusters of dilated lymphatic channels (1), and the necessity of such investigation should be individualized at the discretion of the attending clinician. Lymphangiography and Lymphoscintigraphy using 99 m Tc-nano colloid are capable of demonstrating the site of fistula, caliber, and the number of fistulous communications (6), hence can be described as important second-line investigations, while taking in to account its clinical value, cost and the availability (1, 6). Rigid cystoscopy examination helps in lateralizing chyluria by demonstrating chyle efflux from the ureteric orifice. Although less specific, retrograde pyelogram may be of help in identifying the site of leakage in the presence of chyluria before endoscopic sclerotherapy and during followup.

Symptomatic patients with severe chyluria and malnutrition need intervention rather than a conservative approach (1, 5). Endoscopic sclerotherapy is a minimally invasive treatment option for chyluria. Various types of sclerosant agents such as 0.1-1% silver nitrate, 0.2-5% povidone-iodine, 50% dextrose, 3% hypertonic saline and contrast media can be used for this purpose (1, 6). Initially, sclerosant induced chemical lymphangitis and oedema temporarily block the channels with eventual fibrosis to permanently occlude and close them (4, 5).

Sabinis (1992) pointed out 1% silver nitrate sclerotherapy as a safer and minimally invasive treatment option with a success rate of 82.25% (4). Dhabalia (2010) compared the efficacy of the 3-instillation regime with the 9-instillation regime of 1% silver nitrate in 60 patients with no significant difference in the success rate and concluded that a shorter regime is far better from the standpoint of cost-effectiveness and shorter hospital stay (5). In the absence of a clear consensus regarding the accepted regime, we instilled 10 ml of 0.5% silver nitrate, which was repeated three times in thirty-minute intervals without significant complications.

Sclerosant therapy has its drawbacks about side effects, out of which nausea, vomiting, flank pain, and haematuria are by far the commonest but transient (4, 5). However, high concentration silver nitrate therapy is known to cause dangerous and possibly fatal acute necrotizing ureteritis with obstructive uropathy (6).

10-40% of patients may develop recurrence following sclerotherapy requiring the procedure to be repeated (1). Those patients who develop recurrence within a short period are considered poor responders to repeat sclerotherapy (1). However, our patient was symptom-free for twenty-two months of followup in the clinic.

Failure of endoscopic instillation sclerotherapy requires a pyelo-lymphatic disconnection procedure as a final therapeutic option.

## 4. Conclusion

Chyluria is a rare clinical presentation demanding a high index of clinical suspicion, and symptomatic patients need surgical intervention to relieve symptoms. In this clinical scenario, the assumption of chyluria of nonparasitic origin is valid reasoning in accordance with the detection of the low prevalence of lymphatic filariasis in Sri Lanka and the announcement of the eradication of lymphatic filariasis based on WHO criteria (7). As a corollary, investigating for an uncommon primary cause for nonparasitic chyluria is quite relevant before labeling the condition as idiopathic. Endoscopic sclerotherapy considered a safer and more effective minimally invasive treatment option for severe symptomatic chyluria.
